# Intragenic *MBD5* familial deletion variant does not negatively impact *MBD5* mRNA expression

**DOI:** 10.1186/s13039-014-0080-9

**Published:** 2014-11-19

**Authors:** Sureni V Mullegama, Sarah H Elsea

**Affiliations:** Department of Molecular and Human Genetics, Baylor College of Medicine, One Baylor Plaza, NAB2015, Houston, TX 77030 USA

**Keywords:** 2q23.1 deletion syndrome, *MBD5*, 5′UTR, Intronic deletion, Gene expression, Familial variant

## Abstract

2q23.1 deletion syndrome is characterized by intellectual disability, speech impairment, seizures, disturbed sleep pattern, behavioral problems, and hypotonia. Core features of this syndrome are due to haploinsufficiency of *MBD5.* Deletions that include coding and noncoding exons show reduced *MBD5* mRNA expression. We report a patient with a neurological and behavioral phenotype similar to 2q23.1 deletion syndrome with an inherited intronic deletion in the 5-prime untranslated region of *MBD5*. Our data show that this patient has normal *MBD5* mRNA expression; therefore, this deletion is likely not causative for 2q23.1 deletion syndrome. Overall, it is important to validate intronic deletions for pathogenicity.

The 2q23.1 microdeletion syndrome (OMIM 156200) is a neurodevelopmental disorder characterized by intellectual disability, severe speech impairment, seizures, disturbed sleep pattern, behavioral problems, microcephaly, hypotonia and short stature [1,2] and is caused by deletions of chromosome 2 involving the 2q23.1 band or mutation in the *methyl-CpG binding protein 5* gene, *MBD5* (OMIM 611472) [2]. MBD5 belongs to the MBD family of proteins, which play critical roles in transcriptional regulation and development. The gene structure contains five non-coding exons at the 5-prime end, followed by 10 coding exons (Figure 1). Two protein isoforms have been described [3]. The first isoform contains 1494 amino acids and is encoded by exons 6–15 (Figure 1). The second isoform is shorter, contains 851 amino acids, and is encoded by exons 6–9. Talkowski *et al*. [2] showed that deletions that include noncoding exons, which do not alter the MBD5 protein coding sequence, show reduced *MBD5* mRNA expression, similar to that observed in individuals with larger 2q23.1 microdeletions encompassing the entire *MBD5* gene. The individuals with these *MBD5* non-coding exonic deletions exhibit most features of 2q23.1 microdeletion syndrome [2].

**Figure 1:**
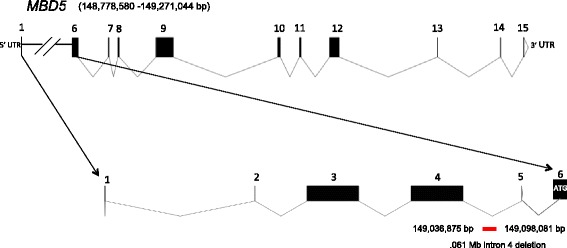
**Intragenic deletion involving a portion of** ***MBD5*** **intron 4** ***.*** A schematic representation of *MBD5,* depicting the 5′-UTR, exons (black boxes), and intronic regions. We depict the .061 Mb intron 4 deletion (in red) in the 5′-noncoding region of *MBD5* at chr(2)(q23.1); (chr2.hg19:g.149,036,875-149,098,081).

In this study, a 4-year-old boy (SMS431) was referred for a possible diagnosis of 2q23.1 deletion syndrome based upon array CGH findings using Nimblegen CGX-12 (Roche Technologies, Penzberg, Germany). The DNA microarray analysis of SMS431, his clinically normal parents, and paternal grandparents was performed in a clinical laboratory, revealing a 0.061 Mb paternally inherited deletion at 2q23.1, arr [hg19] 2q23.1 (149,036,875-149,098,081)x1. This region involves a portion of intron 4 of *MBD5* (Figure 1). This deletion also was found to be present in the paternal grandfather (Figure 2).

**Figure 2:**
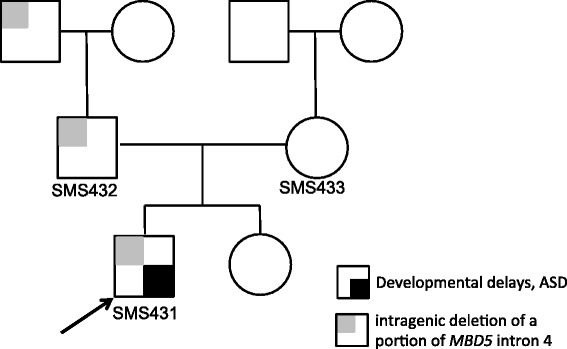
**Pedigree of a family with intron 4 deletion of** ***MBD5.*** The proband (SMS431) is denoted by arrow and small black box. The squares and circles symbolize males and females, respectively. The small light grey boxes denote family members carrying the *MBD5* intron 4 deletion.

While the neurological and behavioral phenotype of SMS431 was remarkably similar to 2q23.1 deletion phenotype (Table 1), it was unclear whether such an intronic deletion in the noncoding region of the *MBD5* gene could be deleterious and manifest the phenotypes present in this child. Additionally, both father and paternal grandfather carry this deletion and neither have features associated with 2q23.1 deletion syndrome or other features observed in this child. Reduced *MBD5* mRNA expression has been reported in 2q23.1 deletion patients and serves as an additional molecular diagnostic indicator for this disorder [2,4]. Consequently, to determine whether a deletion in the noncoding intron 4 region of *MBD5* could result in impaired expression of *MBD5* and was, therefore, responsible for the phenotype observed in SMS431, we evaluated *MBD5* mRNA gene expression on peripheral blood samples obtained from this family.

**Table 1:** **Comparison of the phenotype of SMS431 to the prominent features of 2q23.1 deletion syndrome**

**2q23.1 deletion syndrome**	SMS431*
Developmental delay	+
Motor delay	+
Language impairment	+
Behavioral problems	+
Autistic-like symptoms	+
Sleep disturbances	+
Repetitive behaviors (stereotypies)	+
Self-injurious behaviors	+
Short attention span	+
Aggression	+
Seizures	+
Infantile hypotonia	+
Infantile feeding difficulties	+
Eye abnormalities	+
Heavy arched eyebrows	-
Prominent nose	-
Thin upper lip	+
Widely spaced teeth	-

*Intronic deletion of intron 4 in the 5′UTR region of *MBD5.*

The Institutional Review Board Baylor College of Medicine approved this study. Fresh blood was collected from SMS431 (proband), SMS432 (father), SMS433 (mother), SMS361 (2q23.1 deletion patient) [5], and nine normal controls after informed consent was obtained. Total RNA was isolated according to standard methods (Invitrogen, Carlsbad, CA). RNA was quantified using the NanoDrop ® ND-100 Spectrophotometer and reverse transcribed through qSCRIPT cDNA SuperMix (Quanta Biosciences, Inc., Gaithersburg, MD) according to manufacturer’s instructions. To assess *MBD5* mRNA expression, quantitative RT-PCR was performed as previously described [2,6]. Briefly, Taqman minor groove binder probes for *MBD5* (OMIM 611472, Hs00289233_m1) and *GAPDH* (OMIM 138400, Hs9999905_m1) were used. *GAPDH* was used as the endogenous control. All samples of cDNA were run in triplicate in 10 ul reaction volumes. All samples were run and analyzed according to previously published methods using BioRad CFX Connect™ Real-Time PCR Detection System [2]. Three biological replicates were performed. Results are expressed as fold-change relative to the control sample. Standard error was generated for each sample. A paired t*-*test was used to determine significance. p <0.01 was considered statistically significant.

As expected, *MBD5* mRNA expression for the mother was not statistically different from controls (P = 0.767) (Figure 3), while the 2q23.1 deletion control sample, SMS456, exhibited the typical reduced *MBD5* mRNA expression for an individual with an *MBD5* deletion (P < 0.0001). The mRNA expression levels for the child (SMS431) and his father (SMS432) were not significantly different from controls (P = 0.060) (Figure 3) or from *MBD5* expression for the mother. Further, SMS431 and SMS432 expression patterns were significantly different from a typical 2q23.1 deletion case, SMS456 (P < 0.0001). Since the deletion present in SMS431 and his father did not alter *MBD5* mRNA expression, it is highly unlikely that this intronic deletion affects transcription and translation of *MBD5*, and furthermore, no truncated mRNA, truncated protein or aberrant protein should result.

**Figure 3:**
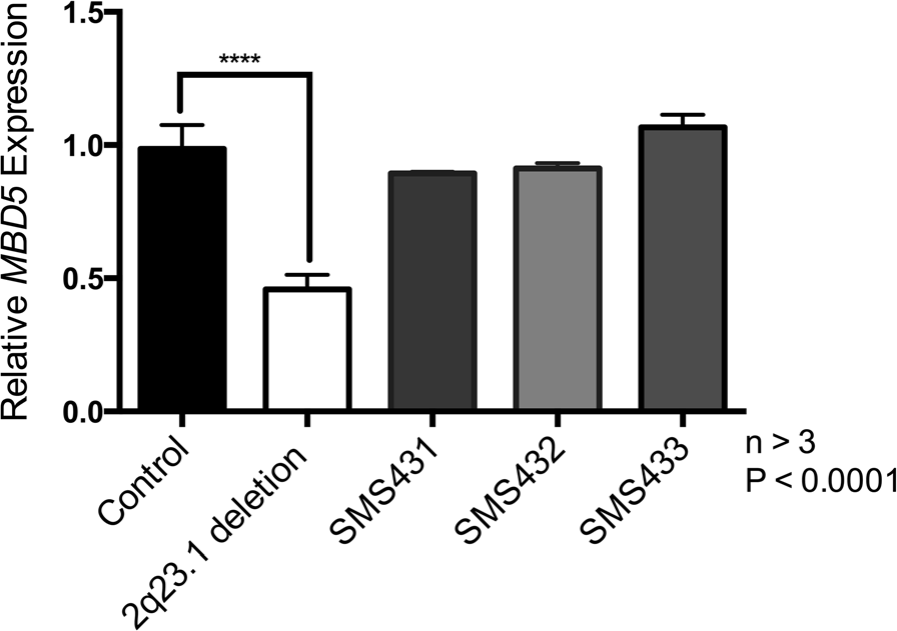
***MBD5*** **mRNA expression is not altered in individuals with** ***MBD5*** **intronic deletion.** Quantitative RT-PCR was used to test *MBD5* mRNA expression levels. *MBD5* mRNA expression levels are not significantly altered in SMS431 (child) or SMS432 (father) or SMS433 (mother) compared to controls. The expression of a typical 2q23.1 deletion syndrome case shows significantly reduced *MBD5* expression compared to controls.

A survey of the Database of Genomic Variants (DGV) contains no deletions that are identical to that observed in the individuals in this family. DVG lists 14 small deletions in the 5′-noncoding region of *MBD5*, with nine deletions confined to intronic sequences, which were reported to be nonpathogenic or of unknown significance. Five of the nine small deletions were found in intron 4.

Overall, it is apparent that the intron 4 deletion does not affect expression of *MBD5* and likely does not give rise to the features associated with the *MBD5* haploinsufficiency that is observed in 2q23.1 deletion syndrome.

Introns in the noncoding region of a gene can play a major role in the transcriptional regulation of a gene and consequently, gene expression. An intron can enhance gene expression through the presence of transcriptional regulatory elements or through structural modulation and splicing [7]. Copy number variants within splice site sequences at the intron-exon junction cause approximately 10% of disease-causing mutations [8]. There are several cases in the medical literature of pathogenic intronic deletions such as *NRXN1* deletions (Autism Spectrum Disorder) [9], *SLC34A3* deletions (hereditary hypophosphatemic rickets with hypercalciuria) [10,11], *PKD1* deletions (Rothmund-Thomson syndrome) [12], and *NASE* deletions (5-fluorouracil toxicity) [13].

Since the intronic deletion identified in SMS431 does not delete the splice junctions between exon 4 and exon 5 or create new splice junctions, proper mRNA splicing likely still occurs, without impacting expression. Further, deletions in 5-prime UTR introns could lead to altered expression if transcription factor binding is affected. Whether there are transcription factors, enhancers, or silencers that bind to that specific intronic region of *MBD5* remains unclear.

In summary, SMS431 exhibited developmental delay, motor delay, severe language impairment, sleep disturbances and behavioral problems that mimicked 2q23.1 deletion syndrome (Table 1). However, these neurological and behavioral phenotypes are observed in other neurodevelopmental disorders. The 2q23.1 deletion syndrome phenotype overlaps with other neurodevelopmental disorders, including Angelman syndrome, Smith-Magenis syndrome, Pitt-Hopkins syndrome, and Kleefstra syndrome [2,6]. SMS431 likely has a different neurodevelopmental disorder that overlaps phenotypically with 2q23.1 deletion syndrome. Since no other copy number variants were identified for this patient, additional testing will be required to determine a cause for the phenotype. The possibility exists that a gene that is involved in a pathway common to MBD5 may be affected, including a gene downstream of MBD5 or binding partner of MBD5. Finally, whole exome sequencing (WES) may be the best approach toward diagnosis for this child.

In conclusion, we highlight the importance of validating intronic deletions for pathogenicity so that accurate and informed diagnosis can be provided to the patient.

## Consent

Written informed consent was obtained from the patient’s parents for publication of this paper. A copy of the written consent is available for review by the Editor-in-Chief of this journal.
